# Programmed Cell Death: Complex Regulatory Networks in Cardiovascular Disease

**DOI:** 10.3389/fcell.2021.794879

**Published:** 2021-11-26

**Authors:** Liuhua Zhou, Jiateng Sun, Lingfeng Gu, Sibo Wang, Tongtong Yang, Tianwen Wei, Tiankai Shan, Hao Wang, Liansheng Wang

**Affiliations:** Department of Cardiology, The First Affiliated Hospital of Nanjing Medical University, Nanjing, China

**Keywords:** cardiovascular diseases, programmed cell death, apoptosis, necrosis, autophagy, pyroptosis, ferroptosis, drug therapy

## Abstract

Abnormalities in programmed cell death (PCD) signaling cascades can be observed in the development and progression of various cardiovascular diseases, such as apoptosis, necrosis, pyroptosis, ferroptosis, and cell death associated with autophagy. Aberrant activation of PCD pathways is a common feature leading to excessive cardiac remodeling and heart failure, involved in the pathogenesis of various cardiovascular diseases. Conversely, timely activation of PCD remodels cardiac structure and function after injury in a spatially or temporally restricted manner and corrects cardiac development similarly. As many cardiovascular diseases exhibit abnormalities in PCD pathways, drugs that can inhibit or modulate PCD may be critical in future therapeutic strategies. In this review, we briefly describe the process of various types of PCD and their roles in the occurrence and development of cardiovascular diseases. We also discuss the interplay between different cell death signaling cascades and summarize pharmaceutical agents targeting key players in cell death signaling pathways that have progressed to clinical trials. Ultimately a better understanding of PCD involved in cardiovascular diseases may lead to new avenues for therapy.

## Background

The development and homeostasis of multicellular organisms depend not only on the regulation of cell survival and renewal, but also on the processing of those cells that are no longer needed or pose a potential danger to the organism. This includes the removal of cells at risk of neoplastic transformation or those infected by pathogens (Kramer et al., 2012; Saunders et al., 2015; Zhu and Sun, 2018). Programmed cell death (PCD) is the primary approach organisms adapt to eliminate these abnormal cells, which stimulates membrane-bound and cytoplasmic proteins by developmental programs and stress-induced signaling, triggering cell death through complex cascade transcriptional and post-translational protein modifications (Shi et al., 2017). Several types of PCD have been discovered in the past 3 decades: autophagy, necrosis, ferroptosis, apoptosis, and pyroptosis (Bai et al., 2017). Each type of PCD has its unique characteristics. Apoptosis represents the coordinated disassembly of dead cells and typical “immune silencing” clearance, while pyroptosis and necrosis refer to the relatively “violent” type of cell death, characterized by the rupture of dead cells, from which effective inflammatory inducers are released (Kung et al., 2011). Autophagy is an evolutionarily conserved catabolic process that begins with forming autophagosomes, a double-membrane-bound structure surrounding cytoplasmic macromolecules and organelles which are ultimately recycled (Nishida and Otsu, 2008; Oka et al., 2012). In addition, another novel iron-dependent PCD mode, ferroptosis, is mainly caused by the peroxidation of unsaturated fatty acids highly expressed on the cell membrane under the action of divalent iron or ester oxygenase inducing cell death (Dixon et al., 2012; Ma et al., 2017). At present, research on PCD is generally limited to a single type, and reports on synergistic or antagonistic effects between different types of PCD are rare.

At present, increasing studies suggest that PCD is a significant cause of cardiovascular diseases such as ischemia/reperfusion (I/R) injury, myocardial infarction (MI) and cardiomyopathy (Tower, 2015; Yang et al., 2016). Each PCD plays a vital role in maintaining cellular homeostasis. Apoptosis ensures the normal development and tissue homeostasis of mature organisms, as well as pyroptosis, ferroptosis, and necrosis protect the host from pathogens and other external threats (Kung et al., 2011; Dixon et al., 2012; Corbalan et al., 2016; Vande Walle and Lamkanfi, 2016). Unlike apoptosis and other PCD, autophagy may commit suicide by undergoing cell death and responding to excessive stress. This view is supported by the analysis of different PCD pathways (Sciarretta et al., 2012). Increasing evidence indicates that different PCD pathways in cardiovascular diseases are associated and interplayed at multiple levels. Still, no article had made a systematic study and report on this issue.

Moreover, many clinical trials have found that the application of appropriate drugs could target cell death signaling pathways in cardiovascular diseases. A clinical phase 2 trial of simvastatin found that targeting inhibition of apoptosis through miR-15a-5p could protect the myocardium in non-coronary surgery, and another trial of berberine found that it could regulate myocardial autophagy through AMPK/mTOR pathway to promote myocardial protection in postoperative patients (Qing et al., 2018; Zhou et al., 2018). However, all the existing studies focus on the targeted therapy through a single type of PCD signaling pathway in cardiovascular diseases. Hitherto, there are no relevant reviews systematically analyzing and collating the interplay of different drugs in cardiovascular diseases through crosstalk between different types of PCD signaling pathways.

Here, we discuss the role of PCD in cardiovascular disease, the interactions between different PCD signaling cascades, and describe the application of drugs targeting cell death signaling pathways in cardiovascular pathology. Moreover, we summarize the latest progress on the relevant studies that have progressed to clinical trials and put forward a novel understanding for the better transformation of PCD pathways in cardiovascular medicine.

## Different Types of PCD and Molecular Mechanisms in Cardiovascular Diseases

### Apoptosis Pathways in Cardiovascular Diseases

**FIGURE 1:**
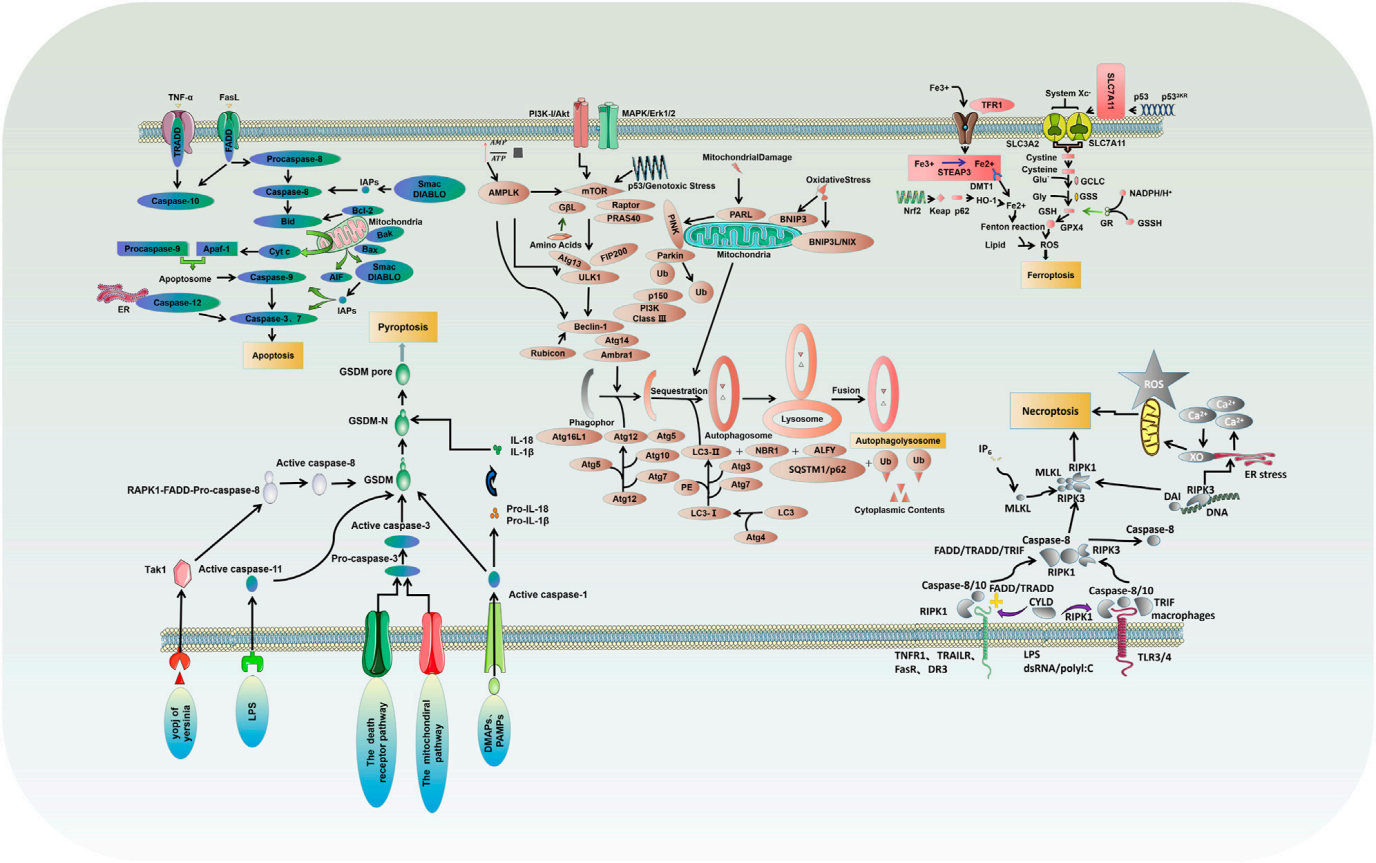
Molecular mechanisms of different types of PCD: critical molecular pathways of autophagy, apoptosis, necrosis, pyroptosis and ferroptosis.

Apoptosis is usually induced by intrinsic and extrinsic (also known as death receptor-mediated) triggers. The intrinsic pathway, also called the mitochondrial or Bcl-2-mediated pathway, is activated by Bh3 proteins (Bim, Puma, Bid, Bmf, Bad, Hrk, Bik, Noxa) to initiate apoptosis when facing intracellular stress caused by various physicochemical factors (Dong et al., 2019). Activated Bax and Bak then form oligomers that promote the activation of the caspase cascade by releasing downstream apoptotic factors such as cytochrome c and Smac/DIABLO from mitochondria, resulting in ultimate protein cleavage and cell death. Extrinsic pathway, also known as death receptor pathway, is the binding and activation of death receptors such as tumor necrosis factor receptor (TNFR) superfamily members, Fas/Fas ligand (FasL) system members and death receptor family members (DR3, DR4 and DR5) with corresponding ligands to form intracellular death-inducing signaling complexes, which activate caspase-8 and its effector (Wang et al., 2021a). However, in normal cells, the Bcl-2 protein family (Bcl-2, Bcl-xl, Mcl-1, Bcl-W, and A1/BFL1) protects cell survival by inhibiting Bax and Bak (Figure 1).

It has been found that myocardial apoptosis can simultaneously exert detrimental effects on cardiac function. For instance, Wu et al. found that bradykinin (BK) could reduce myocardial apoptosis by reducing caspase-3 expression in a human cardiac c-Kit + progenitor cell (hCPCs) myocardial infarction model, thereby improving ischemic heart disease (Wu et al., 2021a), Uberti F et al. showed that levosimendan could interfere with mitochondrial function by regulating mitoK (ATP) channels and NO, and could control the interaction between autophagy and apoptosis to protect H9c2 cells against oxidative damage (Uberti et al., 2011), Nie et al. reported that lncRNA AK006774 could reduce I/R-induced infarct size and myocardial apoptosis by regulating Bcl-2 expression through miR-448 in a mouse I/R model (Nie et al., 2021), Xu et al. found that GSK-3β upregulated CD47 expression in cardiac tissues after MI by activating NF-κb, and then regulated apoptosis leading to myocardial injury (Xu et al., 2021). Besides, AKT1/GSK3β, NF-κb, and other related signaling molecules can also regulate apoptosis in cardiovascular diseases (Supplementary Table S1) (Shen et al., 2020; Jiang et al., 2021a; Wang et al., 2021a; Jiang et al., 2021b; He et al., 2021; Lu et al., 2021; Sun et al., 2021; Xiong et al., 2021; Xu et al., 2021).

### Necrosis Pathways in Cardiovascular Diseases

Necrosis is a type of PCD characterized by lytic necrosis and could drive inflammation, in which the RIPK1/RIPK3/MLKL axis may play an essential role (Chen et al., 2020; She et al., 2019). Receptor-interacting protein kinase1 (RIPK1) autophosphorylation is activated by RIPK3/MLKL sequence, induces membrane permeability and cell destruction to release cell damage-associated molecular pattern (cDAMP), and finally leads to the occurrence of cell necrosis (Zhu and Sun, 2018). Caspase-8 activation, on the other hand, could inhibit cell necrosis by cleaving RIPK1 and RIPK3 (Figure 1). In their study, M.I. Oerlemans et al. demonstrated that RIPK1, RIPK3, and mixed lineage kinase domain-like (MLKL) expression was up-regulated in the myocardium after reperfusion, and the necrosis inhibitor Necrostatin-1 could reduce the phosphorylation levels of RIPK1 and RIPK3 and the recruitment of MLKL, thus inhibiting cell necrosis and reducing myocardial infarct size (Oerlemans et al., 2012). Currently, I/R models are preferred to elucidate the effect of different factors on cardiomyocyte necrosis through various pathways such as mechanistic target of rapamycin (mTOR), rate-oxygenation (ROX) and MLKL (Supplementary Table S2) (Yang et al., 2018a; Zhu et al., 2018; She et al., 2019; Chen et al., 2020; Jiang et al., 2021c; Han et al., 2021; Liu et al., 2021; Pullaiah et al., 2021; Zhang et al., 2021).

### Ferroptosis Pathways in Cardiovascular Diseases

Ferroptosis, characterized by mitochondrial atrophy, increased membrane density, and reduced mitochondrial cristae, is a new form of PCD discovered recently, which proceeds with cell morphology and function different from the various modes of death described above (Dixon et al., 2012). Ferroptosis is usually executed by compressive lipid peroxidation and inhibited by iron chelators. In recent years, research has gradually unveiled its mystery, and many characteristic molecules are identified: acyl-CoA synthetase long-chain family 4 and lysophosphatidylcholine acyltransferase 3 (ACSL4 and LPCAT3) of membrane lipids susceptible to oxidation, xCT of the glutamate-cystine reverse transport system, glutathione peroxidase 4 (GPX4), and ferroptosis inhibitor protein 1, etc. (Figure 1) (Chen et al., 2019; Li et al., 2019).

Current studies have found that cardiovascular diseases caused by high iron levels are associated with ferroptosis (Wu et al., 2021b). Baba et al. have demonstrated that rapamycin could protect cardiomyocytes from excess iron-induced ferroptosis by reacting with mechanistic targets (Baba et al., 2018). Ferrostatin-1 (Fer-1) is an inhibitor of ferroptosis and can mediate the death of cardiomyocytes and neutrophil recruitment after heart transplantation through the toll-like receptor 4/interferon-β (TLR4/TRIF) signaling pathway (Li et al., 2019). Studies also indicate that other signaling molecules such as NADPH oxidase 4 (NOX4) and GPX4 regulate ferroptosis and thus affect heart disease (Supplementary Table S3). Ferroptosis is increasingly considered a potential cause of cardiovascular morbidity, and inhibition of ferroptosis represents a new strategy for these patients (Liu et al., 2018a; Bai et al., 2018; Chen et al., 2019; Fang et al., 2019; Feng et al., 2019; Fan et al., 2021).

### Autophagy Pathways in Cardiovascular Diseases

Autophagy is a highly conserved catalytic process that leads to the autolysosomal degradation of primary cytoplasmic contents, removing abnormally aggregated proteins and excess organelles. Generally, the activation of autophagy is triggered by undernutrition and oxidative stress (Dong et al., 2019). It is also associated with many other physiological and pathological processes, such as development, differentiation, neurodegenerative diseases, stress, infection, and cancer. Autophagy can be classified into three types: macroautophagy, microautophagy, and partner-mediated autophagy (Moujalled et al., 2021). mTOR is an important regulatory molecule that induces autophagy, and activating mTOR by phosphatidylinositol 3-kinase (PI3K) and mitogen-activated protein kinase (MARK) transduction can inhibit autophagy. In contrast, the negative regulation of mTOR by adenosine monophosphate-activated protein kinase (AMPK) and p53 signal transduction promotes autophagy (Dong et al., 2019; Shi et al., 2020). The formed mTOR complex is extensively linked to the downstream autophagy-related gene (ATG) family to construct the autophagosomes and ultimately complete the process of ubiquitin-like reactions. There is an extensive interaction between autophagy and apoptosis, which can be positively or negatively connected. Some studies have suggested the apoptosis-protective factor Bcl-2 could inhibit Beclin-1 dependent autophagy and function as its anti-autophagy regulatory molecule (Figure 1) (Oka et al., 2012; Grootaert et al., 2015; Dong et al., 2019).

In the normal physiological state of the heart, cardiomyocytes, vascular epithelial cells, and smooth muscle cells, autophagy plays an essential role in organelle function and cell renewal. Autophagy is also activated in response to cardiovascular stress, including I/R and heart failure. Sciarretta S et al. found that in ischemic stress, Rheb, as a GTP-binding protein, could inhibit autophagy by activating mTORC1, thereby aggravating post-ischemic infarct size (Sciarretta et al., 2012). During heart failure and aortic atherosclerosis, miR-212/132, laminar flow and Danqi pills regulated autophagy by modulating autophagy-related pathways and molecules, affecting cardiac function (Supplementary Table S4) (Oka et al., 2012; Shi et al., 2020; Grootaert et al., 2015; Ucar et al., 2012; Yuan et al., 2020; Wang et al., 2021b). As mentioned before, autophagy restricted to an appropriate extent protects against ischemic injury via maintaining cardiomyocyte homeostasis, degrading organelles, or misfolding proteins that produce adenosine triphosphate (ATP). However, it is also reported that overwhelming cardiac autophagy induction may also promote cell death and worsen cardiac function in the setting of severe ischemia. Some studies have found that knockdown of TLR4 or NOX4 in rats with heart failure significantly inhibited autophagy activation, thereby improving cardiac function. In addition, the PI3K/AKT/mTOR pathway is indicated to be involved in regulating the excessive autophagy process to protect cardiomyocytes (Supplementary Table S5) (Xuan and Jian, 2016; Xuan et al., 2017; Liu et al., 2018b; Chen et al., 2019; Zhang et al., 2020).

### Pyroptosis Pathways in Cardiovascular Diseases

Pyroptosis is an inflammatory PCD process that rapidly initiates innate immune responses through pattern recognition receptors (PRRs), and different inflammatory bodies can sense different pathogen-associated molecular patterns and damage-associated molecular patterns (PAMPs and DAMPs) that induce pyroptosis (Zhu and Sun, 2018). Classical pyroptosis induction requires activating caspase-1, which cleaves and activates inflammatory cytokines (IL-1b, IL-18), caspase-1 cleaves and activates Gasdermin D (GSDMD), which is also key to the occurrence of pyroptosis (Figure 1) (Zhang et al., 2018a; Yue et al., 2019).

An increasing number of studies have shown that cardiovascular risk factors can activate nod-like receptor protein 3 (NLRP3) inflammasomes in cells. In addition, NLRP3 inflammasomes mediated pyroptosis was observed in various cardiovascular diseases. Studies suggested that NLRP3 inflammasomes and related pyrogenic signaling molecules played an essential role in the progression of cardiovascular diseases (Supplementary Table S6) (Liu et al., 2013; Jeyabal et al., 2016; Zhou et al., 2016; Huang et al., 2017; Nazir et al., 2017; Zhang et al., 2018a; Yang et al., 2018b; Li et al., 2018; Yue et al., 2019; Ding et al., 2020; Li et al., 2020). For example: in myocardial I/R model mice, Ding et al. found that Mi-29a expression was up-regulated and Sirtuin-1 (SIRT1) expression was down-regulated, which in turn regulated NLRP3 to attenuate myocardial I/R injury and enhanced cardiomyocytes survival (Ding et al., 2020).

### Interconnections Between Different PCD Pathways

**FIGURE 2:**
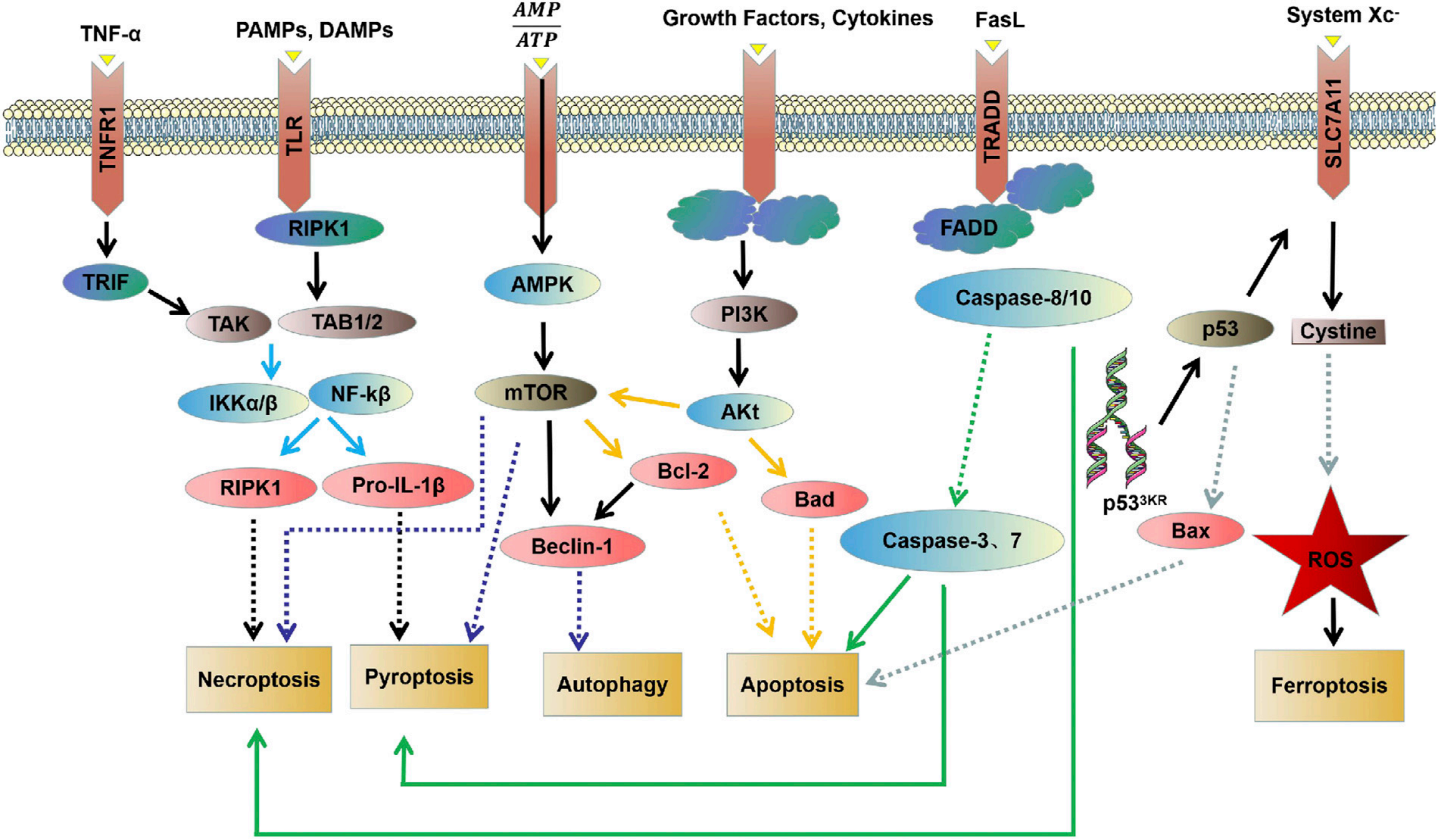
The interrelationship between different PCD molecular mechanisms.

There is now increasing evidence that different PCD pathways in cardiovascular diseases are interconnected and affect each other on multiple levels. Studies have found that the PI3K/AKT pathway can regulate autophagy by targeting mTOR, and can also regulate apoptosis through the Bad pathway (Liu et al., 2018b; Wu et al., 2021a). mTOR kinase is an important regulatory molecule that induces PCD. Studies have found that the AMPK/mTOR pathway is not only involved in necrosis and pyroptosis, but can also regulate autophagy through Beclin-1 (Alers et al., 2012; Nazir et al., 2017; Han et al., 2021). Caspase-3 and caspase-7 act as executors and directly degrade structural and functional proteins, whereas caspase-8 and caspase-10 act as initiators and cause the caspase cascade after being activated by signaling stimuli (Kurokawa and Kornbluth, 2009). The caspase family is involved in the regulation of apoptosis and mediates necrosis and pyroptosis (Martin and Henry, 2013; Galluzzi et al., 2017; Yuan et al., 2018). Wu C et al. found that BK effectively improves cardiac function by reducing cardiomyocyte apoptosis, inflammatory infiltration, and myocardial fibrosis in the infarcted heart by reducing cleaved caspase-3 expression (Wu et al., 2021a). The study carried by Wang K et al. showed that miR-874 could regulate cardiomyocytes necrosis by affecting caspase-8 activity (Wang et al., 2013). Zheng X et al. found that BNIP3 mediated Doxorubicin-induced cardiomyocyte pyroptosis through activation of caspase-3 and cleavage of gasdermin D (GSDMD) (Zheng et al., 2020). In addition, it has been found that there is an interaction between TNF-α/TNFαR, PAMP/TLR, and DAMP/TLR that can activate NF-κB through the IKK pathway. Activation of NF-κb induces the expression of pro-survival genes, including RIPK1 and Pro-IL-1β (Wajant and Scheurich, 2011; Vande Walle and Lamkanfi, 2016). RIPK1 has been implicated in the regulation of necroptosis, whereas Pro-IL-1β mediated pyroptosis (Van Herreweghe et al., 2010; Yuan et al., 2018). An interaction between ferroptosis and apoptosis in cardiovascular diseases was also found in the present study (Figure 2).

## Different Types of PCD and Clinical Drug Therapy

### Targeting Apoptosis in Clinical Practice

Currently, interventions targeting apoptosis may have a significant impact on the treatment of cardiovascular diseases. Bcl-2 family, p53 gene, IEG family, ICE gene family, and Fasl-Fas system play essential roles in initiating and developing apoptosis (Dispersyn and Borgers, 2001). Accumulating evidence from clinical trials suggested that modulation of the Bcl-2 family and the Fasl-Fas system as a novel intervention could improve myocardial function (Corbalan et al., 2016). Zhou et al. and Li et al. reported that statins could regulate the cardiomyocytes apoptosis in patients by regulating the expression of Bcl-2 and Bax, thereby protecting the cardiac function (Zhou et al., 2018; Li et al., 2016); Adamopoulos S et al. and Skudicky D et al. demonstrated that growth hormone (GH) and pentoxifylline could alleviate cardiac insults by modulating the apoptosis of cardiomyocytes and the level of soluble Fasl/Fas system (Adamopoulos et al., 2002; Skudicky et al., 2000). Other agents were also found to achieve the efficacy of myocardial protection in apoptosis-targeted ways (Supplementary Table S7) (Rössig et al., 2001; Zhang et al., 2005; Zhang et al., 2018b; Qing et al., 2018). Considering that apoptosis plays a vital role in cardiovascular pathologies, elucidating the underlying therapeutic mechanisms of these drugs in clinical trials provides us with a brand-new concept and idea for the occurrence and development of cardiovascular diseases.

### Targeting Necrosis in Clinical Practice

Necrosis is a class of cell death initiated by death receptor ligands, mediated through death receptors, occurs in the inhibition of apoptotic pathways. The death receptor-receptor interaction protein-mitochondria-oxygen free radical pathway is proved to be an essential regulator of cardiac necrosis, and current clinical trials have found that drugs targeting this pathway can reduce cellular necrosis and improve cardiac function (Kim et al., 2011; Vandenabeele et al., 2010; Thévenod and Lee, 2013). Verma S et al. verified the effect of PZ-128 in a phase 2 clinical trial. The results indicated that it could reduce cyclic myocardial necrosis by targeting protease-activated receptor-1 (PAR1) on vascular cells on the inner surface of recipient cells, thereby further improving the outcome of patients with myocardial infarction (Wang, 2018); Silvain J et al. validated the combined efficacy of ticagrelor and clopidogrel on patients undergoing percutaneous coronary intervention (PCI) in a phase 3b clinical trial and found that apart from their anti-platelet aggregation and effect, it could also reduce perioperative myocardial necrosis by inhibiting NF-κb pathway and further improve the postoperative recovery of patients with myocardial infarction (Silvain et al., 2020). Other studies found that some drugs could regulate cell necrosis by anti-aggregation, effect or reducing cell calcium overload, thereby improving myocardial necrosis in patients before and after surgery (Supplementary Table S8) (Alexander et al., 2008; Talarico et al., 2009; Lader et al., 2016; Nauck et al., 2018; Liu et al., 2019; Mohammad et al., 2020; Tcheng et al., 2020). At present, more and more attention has been focused on the clinical trials evaluating anti-necrosis therapies in cardiovascular diseases. The deepening of its targeted treatment in cardiovascular diseases will undoubtedly profoundly impact the clinical treatment of cardiovascular diseases.

### Targeting Ferroptosis in Clinical Practice

Unlike classical apoptosis, there is no hallmark of apoptosis such as chromatin condensation during ferroptosis, but it is accompanied by mitochondrial shrinkage and accumulation of lipid oxides. Studies have revealed that overloaded iron would cause irreparable damage to multiple organs. The use of inhibitors of apoptosis in clinical trials does not also inhibit ferroptosis, while the use of iron chelators can inhibit the ferroptosis process of cells. Wongjaikam S et al. demonstrated that a combination of lamotrigine and desferrioxamine as chelation therapy could reduce the level of myocardial iron by restoring sarco/endoplasmic reticulum calcium ATPase (SERCA) levels (Wongjaikam et al., 2017). Farmaki K et al. also found that the combined chelation therapy of desferrioxamine and desferrioxone can prevent and reverse the cardiac complications caused by excessive iron in blood transfusion (Farmaki et al., 2010). Other clinical trials concerning iron chelators in cardiovascular diseases are shown in Supplementary Table S9 (Penckofer and Schwertz, 2000; Al-Rousan et al., 2009; Farmaki et al., 2010; Lal et al., 2013; Jansová et al., 2014; Shakoor et al., 2014; Pennell et al., 2015; Ho et al., 2017; Eghbali et al., 2019). Although iron ion chelators are manifested as a promising drug target in clinical trials, their potential molecular signaling pathways and networks remain to be explored. Besides, the metabolism of iron at the systemic level is another challenge. Iron metabolism forms homeostasis between the body and target organs to regulate iron death in the clinical treatment of cardiovascular diseases.

### Targeting Autophagy in Clinical Practice

Autophagy plays a broad role in developing cardiovascular pathology and can act as both a suppressor and a promoting factor of cardiovascular disease. Although some drugs and regimens can modulate autophagy, few clinical trials have assessed their role in the cardiovascular system. Among them, Qing et al. conducted a phase 2 clinical trial of berberine and found that it could reduce myocardial injury by regulating the AMPK/mTOR pathway; Hua et al. elucidated that simvastatin could reduce myocardial injury by regulating the expression of LC3-II/LC3-I, Beclin- 1, and AMPK phosphorylation, which in turn improved cardiac function (Supplementary Table S10) (Hua et al., 2017; Qing et al., 2018). Another significant limitation in this field is the lack of spatially specific delivery of exogenous autophagy modulators for cardiac or vascular repair, so there is still a long way to go for effective autophagy therapy. However, the insights gained from these works will inspire future clinical trials assessing autophagic responses to achieve therapeutic efficacies.

### Targeting Pyroptosis in Clinical Practice

Pyroptosis is a new programmed inflammatory death mainly regulated by classical and non-classical pathways, the classical pathway of which is mediated by caspase-1 dependence, and the non-classical inflammasome pathway is mediated by caspase-4/5/11 (Li et al., 2020). Numerous studies have shown that pyroptosis is generally involved in the development and outcome of various cardiovascular diseases such as myocardial infarction, heart failure, and myocardial I/R (Zhu and Sun, 2018; Wang and Kanneganti, 2021). To date, few clinical trials have assessed its role in cardiovascular disease, mainly due to the lack of cell/organ-targeted delivery of exogenous pyroptosis modulators for cardiac or vascular repair. However, since pyroptosis is involved in the development of a variety of cardiovascular diseases, the subsequent in-depth study of cell/organ targeted delivery of apoptosis modulators will provide a new direction and effective target for the therapeutic research of related diseases.

## Summary and Discussion

While research into cardiovascular disease has focused on the molecular mechanisms of individual PCD types, it is now clear that different PCD pathways do not operate in isolation. Indeed, a more likely explanation basing on current knowledge about the various possibilities of triggering and reconnecting PCD signaling cascades suggests that autophagy, apoptosis, ferroptosis, pyroptosis, and necrosis constitute a pluralistic, coordinated cell death system in which one pathway can flexibly compensate for the other (Konstantinidis et al., 2012; Tower, 2015; Wang and Kanneganti, 2021). For example, it is demonstrated that in I/R injury models, both pyroptosis and necrosis could affect cardiovascular disease through AMPK/mTOR and other pathways (Oerlemans et al., 2013; Nazir et al., 2017). In this review, we describe the characteristics and molecular mechanisms of individual PCD types and discuss the interactions between different cell death signaling cascades to provide a better understanding of the relationship between PCD and cardiovascular diseases.

**FIGURE 3:**
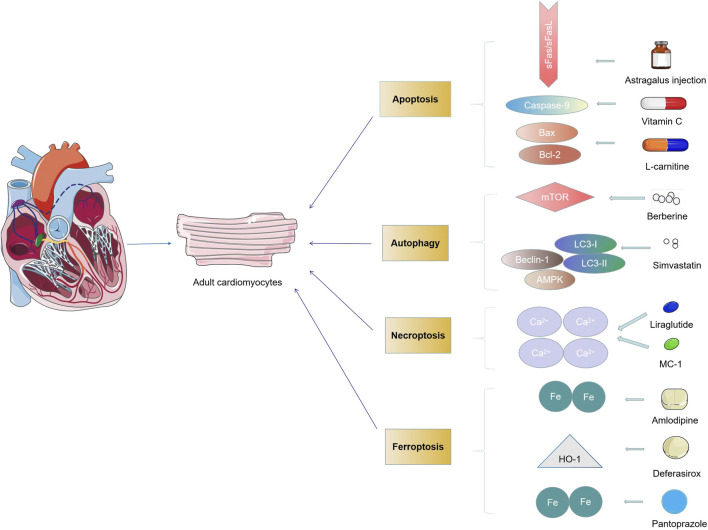
Drugs target cardiomyocytes through different types of PCD.

Up to now, while the increasingly important role of different PCD is unveiled, few clinical trial studies are conducted to treat cardiovascular diseases by pharmacological intervention to PCD (Mani, 2008). Therefore, we describe the critical role of drugs targeting cell death signaling pathways and their efficacy in various cardiovascular diseases by collecting currently available drugs studied in clinical trials. Multiple medications are targeted in cardiovascular disease by modulating different types of PCD in many clinical trials. For instance, statins can regulate myocardial apoptosis in patients by regulating the expression of Bcl-2 and Bax (Zhou et al., 2018). Simvastatin can regulate the autophagy of cardiomyocytes by regulating the expression of LC3-I/II, Beclin-1, and other factors (Hua et al., 2017). Drugs such as liraglutide and pyridoxal 5′-phosphate (MC-1) can modulate the necrosis of cardiomyocytes by intervening calcium ions (Nauck et al., 2018; Alexander et al., 2008). Amlodipine and pantoprazole regulate ferroptosis in cardiomyocytes by affecting iron absorption (Shakoor et al., 2014; Eghbali et al., 2019) (Figure 3). Therefore, more work is needed to explore whether drugs can target therapy in various cardiac cells by handling multiple types of PCD. Most previous drug studies reported only a single PCD type, while recent studies found that drugs could act simultaneously by regulating multiple different types of PCD. For example, Qing et al., in phase 2 of clinical trials, found that berberine could reduce cardiomyocyte autophagy and apoptosis by regulating the AMPK/mTOR pathway, thereby reducing myocardial injury in PCI patients (Qing et al., 2018). In general, treatment for any type of PCD can reverse further damage to the heart to some extent (Moujalled et al., 2021). More research is needed to clarify whether PCD acts as an initiator or a critical node in the injury path when studying drugs acting through the PCD pathway.

Apart from cardiovascular disorders, PCD is also involved in other diseases, including respiratory and digestive tumors (Venderova and Park, 2012; Tai et al., 2018). However, current studies reported only PCD on a single type of cell, and the effects and differences between different types of cells are poorly informed. Therefore, in future work, we can start from various cell types and study the impact of drugs on cardiovascular diseases by intervening in various types of PCD in different cell types, which can not only reduce the adverse events of PCD-targeted therapy but also more accurately treat various types of cardiovascular diseases.

While the underlying biological mechanisms regarding the PCD remain uncovered, therapeutic manipulation of cell death pathways holds great promise. At present, among various types of diseases, the most studied is the intervention of drugs in various tumors: recent clinical trials reported that the combination of deferoxamine (DFO) and deferiprone (DFP) effectively improves hematologic malignancies (Wongjaikam et al., 2017). The combined therapy of drugs regulates ferroptosis in the clinical treatment of various diseases by affecting iron metabolism at the systemic level and allowing iron metabolism to form homeostasis between the body and target organs. Based on this, recently, Farmaki K et al. found that combination therapy with desferrioxamine drugs also improved prognosis in cardiovascular disease and has been initiated in clinical trials (Farmaki et al., 2010). Moreover, combination therapy with DFO and DFP can improve cardiac function by inhibiting ferroptosis by reducing iron levels in the heart (Wongjaikam et al., 2017). Studies in future work can be carried out in different types of cardiovascular diseases based on drug-mediated signaling pathways.

Therefore, our review may pave the way for further novel research in the future. On the one hand, we hope to uncover complex signaling pathways between different PCD and clarify their functions in cardiovascular pathologies. On the other hand, acceleration in clinical transformation is warranted to enable PCD-targeted agents to be applied to detect, prevent, and treat cardiovascular diseases.
