# Supplementary material for: Programmed Cell Death: Complex Regulatory Networks in Cardiovascular Disease
Source: Front Cell Dev Biol. 2021 Nov 26;9:794879. doi: 10.3389/fcell.2021.794879 (PMC8661013; doi:10.3389/fcell.2021.794879)
Supplement: Supplementary file 1 [file Table1.DOCX]

| Reagents | Materials | Diseases | Mechanisms | Effects | Reference |
| --- | --- | --- | --- | --- | --- |
| Long non-coding RNA NORAD | Rat | MI | Via miR-577/COBLL1 axis | Aggravate acute MI by promoting apoptosis | [31] |
| NDP52 | Mice | MI | Through interaction with TBK1 and RAB7, leading to RAB7 phosphorylation,thus preventing cardiomyocyte apoptosis in MI | Promote cardioprotection against ischemic heart diseases | [32] |
| BK | Rat | MI | By promoting pAkt and Bcl-2 expression and reducing cleaved caspase 3 and Bax expression to decrease cardiomyocyte apoptosis | Improve cardiac function | [27] |
| AZA | Rat | I/R | By AKT1/GSK3β pathway to reduce cardiomyocyte apoptosis | Ameliorate myocardial I/R injury in diabetic rats | [33] |
| Long non-coding RNA AK006774 | Mice | I/R | By AK006774/miR-448/bcl-2 signaling axis to antagonize the effects of AK006774 on cardiomyocyte apoptosis | Inhibit cardiac I/R injury | [29] |
| GSK-3β | Rat | MI | By activating NF-kB | Lead to cardiomyocytes damage and apoptosis | [30] |
| CTRP13 | Rat | I/R | Via regulating the AMPK/Nrf2/ARE signaling pathway | Attenuate myocardial I/R injury | [34] |
| LncRNA Chaer | Mice | MI | Through AMPK activation | Prevent cardiomyocyte apoptosis from acute MI | [20] |
| Huoxin pill | Mice | MI | Via suppression of p53 and TGF-β1/Smad2/3 pathways | Attenuate MI-induced apoptosis | [35] |
| Krüppel-like factor 15 | Mice | MI | By regulating p38/MAPK signaling | Reduce ischemia-induced apoptosis | [26] |
| Long non-coding RNA LINC00261 | Mice | MI | By LINC00261/miR-522-3p/TNRC6A | Increase the viability of cardiomyocytes and inhibit apoptosis | [36] |

Table 1: Possible mechanisms by which apoptosis inducers regulate apoptosis in the treatment of cardiovascular diseases. (NDP52: Nuclear dot protein 52, BK: Bradykinin, AZA: Azathioprine, GSK-3β: Glycogen synthase kinase 3 beta, CTRP13: C1q/TNF-related protein 13, MI: Myocardial infarction, I/R: Ischemia/reperfusion, COBLL1: Cordon-bleu-like 1, TBK1: Trichobakin1, RAB7: Ras-related in brain 7, pAKT: phospho-Akt, AMPK: Adenosine monophosphate-activated protein kinase, Nrf2: Nuclear factor (erythroid-derived 2)-like 2, ARE: Antioxidant response element, TGF-β: Transforming growth factor beta, MAPK: Mitogen-activated protein kinase, TNRC6a: Trinucleotide repeat-containing gene 6a.）
