# Supplementary material for: Programmed Cell Death: Complex Regulatory Networks in Cardiovascular Disease
Source: Front Cell Dev Biol. 2021 Nov 26;9:794879. doi: 10.3389/fcell.2021.794879 (PMC8661013; doi:10.3389/fcell.2021.794879)
Supplement: Supplementary file 2 [file Table8.DOCX]

| Drug | Diseases | Trial  Phase | Sample  Size | Mechanisms | Effects | NCT | Reference |
| --- | --- | --- | --- | --- | --- | --- | --- |
| PZ-128 | Patients With With or Without T2DM History of MI or Stroke | Ⅱ | 100 | Inhibit PAR1 on platelets and other vascular cells by targeting the intracellular surface of the receptor | Reduce periprocedural myonecrosis | NCT02561000 | [90] |
| liraglutide | Patients with MI | Ⅱ | 9340 | Through Homer1-dependent regulation of ER calcium homeostasis | Reduce myocardial necrosis following regional ischaemia induction | NCT01179048 | [92] |
| CMX-2043 | Patients undergoing elective PCI | Ⅱ | 142 | By regulating the Akt pathway | Reduce PCI periprocedural myonecrosis | NCT00984802 | [93][98] |
| MC-1 | Patients undergoing high-risk coronary artery bypass graft surgery | Ⅲ | 3023 | By preventing cellular calcium overload | Reduce myocardial necrosis | NCT00402506 | [94] |
| Ticagrelor | Patients after PCI | Ⅲ | 1910 | Reduce periprocedural ischaemic complications/by inhibiting the NF-kB pathway | Reduce periprocedural myocardial necrosis | NCT02617290 | [91][96] |
| Tirofiban | Patients after PCI | Ⅱ | 46 | By improving microvascular perfusion | Reduce post PCI myonecrosis and improve coronary flow in diabetics | None | [95] |
| Clopidogrel | Patients after PCI | Ⅲ | 1910 | Via anti-aggregatory and anti-inflammatory effects | Reduce periprocedural myocardial necrosis | NCT02617290 | [91][97] |

Table 8: Clinical trials involving necrosis of the cardiovascular system. (PZ-128: Protease-activated receptor 1 inhibitor, CMX-2043: r-lipoyl-l-glutamyl-l-alanine, MC-1: pyridoxal 5´-phosphate, T2DM: Type 2 Diabetes mellitus, PCI: Percutaneous coronary intervention, MI: Myocardial infarction, PAR1: Protease-activated receptor 1, ER: Endoplasmic reticulum, Akt: Serine-threonine kinase. )
