# Supplementary material for: Programmed Cell Death: Complex Regulatory Networks in Cardiovascular Disease
Source: Front Cell Dev Biol. 2021 Nov 26;9:794879. doi: 10.3389/fcell.2021.794879 (PMC8661013; doi:10.3389/fcell.2021.794879)
Supplement: Supplementary file 3 [file Table7.DOCX]

| Drug | Diseases | Trial  Phase | Sample  Size | Mechanisms | Effects | NCT | Reference |
| --- | --- | --- | --- | --- | --- | --- | --- |
| Simvastatin | Patients undergoing noncoronary cardiac surgery | Ⅱ | 70 | Via suppressing miR-15a-5p expression, leading to increasing expression of Bcl-2 and decreasing expression of Bak | Protected the myocardium in patients undergoing noncoronary artery cardiac surgery | None | [23] |
| L-carnitine | patients undergoing valve replacement under cardiopulmonary bypass | Ⅱ | 90 | Through modulating the expressions of Bcl-2 and Bax | Reduce cardiopulmonary bypass-induced myocardial apoptosis, resulting in a protective effect from MIRI | None | [81] |
| Iohexol | CAD patients with DM | Ⅱ | 100 | Induce EMP release and inhibited the viability and induce apoptosis of HUVECs, as well as increasing Bax and cleaved caspase-3 and decreasing Bcl-2 | Less endothelial apoptosis | ChiCTR-TRC-14005183 | [84] |
| Vitamin C | CHF patients | Ⅱ | 34 | Reduce cytochrome C release from mitochondria and the inhibition of caspase-9 activity | Inhibit endothelial cell apoptosis in congestive heart failure | None | [85] |
| Berberine | Patients after PCI | Ⅱ | 100 | Through the AMPK/mTOR pathway | Reduce myocardial injury | CMU-201500605 | [24] |
| GH | Patients with CHF secondary to idiopathic dilated cardiomyopathy | Ⅱ | 10 | Reduce serum levels of proinflammatory cytokines and soluble Fas/FasL system | Improve in clinical performance and exercise capacity of patients with IDC | None | [82] |
| Pentoxifylline. | Patients with idiopathic dilated cardiomyopathy | Ⅱ | 49 | Reduce in Fas/APO-1 plasma concentrations | Improve in left ventricular function in patients with idiopathic dilated cardiomyopathy | None | [83] |
| AI | Patients with CHF | Ⅱ | 72 | By affecting sFas/sFasL | Improve cardiac function in the aged patients with CHF | None | [86] |

Table7:Clinical trials involving apoptosis of the cardiovascular system. (GH:Growth hormone, AI: Astragalus injection, MIRI: Myocardial ischemia-reperfusion injury, CAD: Coronary artery disease, DM: Diabetes mellitus, EMP: Endothelial microparticle, HUVECs: Human umbilical vein endothelial cells, CHF: Chronic heart failure, PCI: Percutaneous coronary intervention, AMPK/mTOR: AMP-activated protein kinase/mammalian target of rapamycin, IDC: Idiopathic dilated cardiomyopathy. )
