# Supplementary material for: Programmed Cell Death: Complex Regulatory Networks in Cardiovascular Disease
Source: Front Cell Dev Biol. 2021 Nov 26;9:794879. doi: 10.3389/fcell.2021.794879 (PMC8661013; doi:10.3389/fcell.2021.794879)
Supplement: Supplementary file 4 [file Table9.DOCX]

| Drug | Diseases | Trial  Phase | Sample  Size | Mechanisms | Effects | NCT | Reference |
| --- | --- | --- | --- | --- | --- | --- | --- |
| Amlodipine | Patients with thalassaemia with moderate-to-severe myocardial iron deposition | Ⅱ | 20 | L-shaped Ca(2+) channels block iron uptake by cardiomyocytes | Reduce myocardial iron | NCT02065492 | [101] |
| DFX | In transfused patients | Ⅱ | 46 | By affecting the formation of highly toxic hydroxyl radicals | Improve cardiac iron load | NCT00673608 | [102][103] |
| DFO | Patients with severe iron overload with thalassemia | Ⅱ | 103 | Reduce cardiac protein oxidation and superoxide abundance,and diminish the phosphorylation of all three MAPK subfamilies | Improvement in myocardial iron | NCT00600938 | [104][105] |
| Pantoprazole | Patients with thalassemia major and intermedia | Ⅱ | 60 | Interrupt iron absorption in conditions with increased iron absorption | Reduce serum ferritin in patients with thalassemia major and intermedia | IRCT2017042220258N40 | [106] |
| HRT | Postmenopausal women | Ⅱ | 54 | Through improved iron status parameters | Decreased cardiovascular risk in postmenopausal women | None | [107] |
| DFO and DFP | Patients receiving treatment for cardiac dysfunction | Ⅱ | 52 | Normalize patients' iron load | Prevent and reverse cardiac complications associated with transfusion iron overload | None | [100] |
| DFO and DFX | Patients with persistent iron overload or organ damage | Ⅱ | 22 | By restoring cardiac sarcoplasmic endoplasmic reticulum Ca2+ ATPASE (SERCA) | Reduce systemic and myocardial iron | NCT00901199 | [108][99] |

Table9: Clinical trials involving ferroptosis of the cardiovascular system. (DFX: Deferasirox, DFO: Deferoxamine, HRT: Hormone replacement therapy, DFP: Deferiprone, MAPK: Mitogen-activated protein kinase, SERCA: Sarcoplasmic/endoplasmic reticulum Ca2+-ATPase. )
