# Supplementary material for: Programmed Cell Death: Complex Regulatory Networks in Cardiovascular Disease
Source: Front Cell Dev Biol. 2021 Nov 26;9:794879. doi: 10.3389/fcell.2021.794879 (PMC8661013; doi:10.3389/fcell.2021.794879)
Supplement: Supplementary file 5 [file Table2.DOCX]

| Reagents | Materials | Diseases | Mechanisms | Effects | Reference |
| --- | --- | --- | --- | --- | --- |
| Nicorandil | Mice | Microcirculation reperfusion injury | Mito-KATP channel-opening effect | Reduce myocardial necrosis | [40] |
| Esculetin | Rat | Isoproterenol induced myocardial toxicity | Reduce intracellular ROS inhibition and mRNA expression of pro-inflammatory cytokines including TNF-α, IL-6 and NF-κB | Prevent necrosis | [41] |
| β-carboline derivative 17c | Rat | I/R | Increase the levels of SOD and GSH-Px | Inhibit the apoptosis of cardiomyocytes and the size of myocardial infarction was significantly reduced after I/R injury in vivo | [42] |
| EA | Rat | I/R | Through the PI3K/Akt/mTOR pathway to reduce necrosis | Alleviate myocardial I/R injury | [43] |
| PKA | Mice | I/R | By mTORC1/p62/Keap1/Nrf2 | Aggravate cardiomyocyte necrosis and myocardial I/R injury | [19] |
| Necrostatin-1 | Mice | I/R | By RIPK1/RIPK3/MLKL axis to inhibit cell necrosis | Reduce the area of MI and reduce the formation of heart scars | [39] |
| Arctiin | Rat | I/R | Via scavenging reactive oxygen species and targeting RIPK1 and/or MLKL to reduce necrosis | Protect rat heart against I/R injury | [37] |
| PGAM5 | Rat | I/R | Through suppression of dynamin-related protein 1 | Reduce necroptosis in rat hearts following I/R | [38] |
| Melatonin | Mice | I/R | Through inhibiting RIP3-MLKL/CaMKII dependent necrosis | Attenuate chronic pain related myocardial ischemicsusceptibility | [44] |
| Ripk3 | Mice | I/R | A mechanism involving calcium overload/XO/ROS/mPTP pathway | Promote ER stress-induced necrosis in cardiac I/R injury | [45] |

Table 2: Possible mechanisms by which necrosis inducers regulate necrosis in the treatment of cardiovascular disease. (EA: Electroacupuncture, PKA: Protein kinase A, PGAM5: Phosphoglycerate mutase 5, Ripk3: Receptor-interacting protein 3, mito-KATP: mitochondrial KATP, ROS: Reactive oxygen species, TNF: Tumor necrosis factor, SOD: Superoxide dismutase, GSH-PX: Glutathione peroxidase, PI3K: Phosphatidylinositol 3-kinase, Akt: Serine-threonine kinase, mTOR: mammalian target of the rapamycin, mTORC1: mammalian target of rapamycin complex 1, RIPK: Receptor-interacting protein kinase, MLKL: Mixed-lineage kinase domain-like, RIP3: Receptor-interacting protein 3, CaMKII: Ca2+/calmodulin-dependent kinase II, XO: Xanthine oxidase, mPTP: mitochondrial permeability transition pore, ER: Endoplasmic reticulum, MI: Myocardial infarction, I/R: ischemia-reperfusion. )
