# Supplementary material for: Programmed Cell Death: Complex Regulatory Networks in Cardiovascular Disease
Source: Front Cell Dev Biol. 2021 Nov 26;9:794879. doi: 10.3389/fcell.2021.794879 (PMC8661013; doi:10.3389/fcell.2021.794879)
Supplement: Supplementary file 6 [file Table5.DOCX]

| Reagents | Materials | Diseases | Mechanisms | Effects | Reference |
| --- | --- | --- | --- | --- | --- |
| MicroRNA-223 | Rat | MI | Via the Akt/mTOR pathway by targeting PARP-1 to inhibite excessive autophagy | Protect neonatal rat cardiomyocytes | [17] |
| TLR4 and NOX4 | Rat | Heart failure | TLR4-NOX4 as a potential therapeutic target for HF through inhibiting autophagy and ferroptosis | Improve left ventricular remodeling and reduce cardiomyocytes death | [22] |
| MHBFC | Rat | I/R | Activate the PI3K/Akt signal pathway to inhibit excessive autophagy | Ameliorate myocardial I/R injury | [59] |
| Dexmedetomidine | Rat | Myocardial ischemia-reperfusion injury | Up-regulate the SIRT1/mTOR axis and decrease overautophagy in I/R rats | Relieve I/R injury, reduce cardiomyocyte apoptosis, oxidative stress and inflammatory reactions | [60] |
| Epigallocatechin gallate | Rat | I/R | Through the PI3K/Akt pathway-mediated inhibition of the restoration of the autophagic flux | Reduce the infarct size, decrease the apoptotic rate and partially preserve heart function | [61] |

Table 5: Possible mechanisms of autophagy inducers in the treatment of cardiovascular disease by down-regulating autophagy. (TLR4: Toll-like receptor 4, NOX4: NADPH oxidase 4, MHBFC: 17-Methoxyl-7-hydroxy-benzene-furanchalcone, mTOR: mammalian target of rapamycin, Akt: Serine-threonine kinase, PARP-1: Poly (ADP)-ribose polymerase-1, SIRT1: Sirtuin 1, MI: Myocardial infarction, I/R: Ischemia/reperfusion. )
