# Supplementary material for: Programmed Cell Death: Complex Regulatory Networks in Cardiovascular Disease
Source: Front Cell Dev Biol. 2021 Nov 26;9:794879. doi: 10.3389/fcell.2021.794879 (PMC8661013; doi:10.3389/fcell.2021.794879)
Supplement: Supplementary file 7 [file Table3.DOCX]

| Reagents | Materials | Diseases | Mechanisms | Effects | Reference |
| --- | --- | --- | --- | --- | --- |
| Fer-1 | Mice | I/R | by promoting adhesion of neutrophils to coronary vascular endothelial cells through a TLR4/TRIF/type I IFN signaling pathway | Reduce infarct size, improve left ventricular systolic function, and reduce left ventricular remodeling. | [46] |
| ENPP2 | H9c2 cell | Hypertrophy | Through modulating GPX4, ACSL4 and NRF2 expression and enhancing AKT survival signal | Protect cardiomyocytes from erastin-induced ferroptosis | [49] |
| TLR4 | Rat | Heart failure | Inhibit ferroptosis-mediated cell death through TLR4/NADPH oxidase 4 pathway | Improve left ventricular remodeling and reduce myocytes death | [22] |
| Puerarin | Rat | Heart failure | Mitigate ferroptosis by regulating the expression of NOX4 and GPX4 | Protect against heart failure induced by pressure overload | [50] |
| miR-15a-5p | Mice | MI | Reduce ferroptosis by regulating Egr-1/miR-15a-5p/GPX4 | Alleviate MI | [51] |
| MitoTEMPO | Mice | Cardiomyopathy | Scavenge lipid peroxidation specifically in the mitochondria | Attenuate DOX-induced cardiomyopathy | [52] |
| Lip-1 | Mice | I/R | Increase GPX4 protein levels and reduce ROS generation | Reduce myocardial infarct size and I/R injury | [53] |
| mTOR | Mice | Hypertrophy | Regulate cellular iron transport and reduce ROS production | Protect cardiomyocytes against excess iron and ferroptosis | [48] |

Table 3: Possible mechanisms by which ferroptosis inducers modulate ferroptosis to treat cardiovascular disease. (Fer-1: Ferrostatin-1, ENPP2: Ectonucleotide pyrophosphatase/phosphodiesterase 2, TLR4: Toll-like receptor 4, miR-15a-5p: microRNA-15a-5p, Lip-1: Liproxstatin-1, mTOR: mechanistic target of rapamycin, TRIF: Toll-interleukine-1 receptor domain-containing adapter-inducing interferon-β, TLR: Toll-like receptor, ACSL4: Acyl-CoA synthetase long-chain family member 4, GPX4: Glutathione peroxidase 4, Nrf2: Nuclear factor erythroid 2-related factor 2, NOX4: NADPH oxidase 4, GPX4: Glutathione peroxidases 4, Egr-1: Early growth response-1, ROS: Reactive oxygen species, I/R: Ischemia/reperfusion, MI: Myocardial infarction. )
