# Supplementary material for: Programmed Cell Death: Complex Regulatory Networks in Cardiovascular Disease
Source: Front Cell Dev Biol. 2021 Nov 26;9:794879. doi: 10.3389/fcell.2021.794879 (PMC8661013; doi:10.3389/fcell.2021.794879)
Supplement: Supplementary file 8 [file Table4.DOCX]

| Reagents | Materials | Diseases | Mechanisms | Effects | Reference |
| --- | --- | --- | --- | --- | --- |
| Rheb | Mice | MI | Ras homolog enriched in brain,a GTP-binding protein that inhibits autophagy by activating mTORC1 | Exhibit greater cardiac damage on MI | [15] |
| MiRNA-212/132 | Mice | Heart failure | Be associated with improved FOXO3-dependent autophagic responses | Protect mice from heart failure caused by pressure overload | [57] |
| Dnase2a-/- | Mice | Heart failure | Lack a lysosomal DNAse involved in the autophagic degradation of mitochondrial DNA released on organelle damage | Develope severe myocarditis and dilate cardiomyopathy associated with premature death after treatment with pressure overload | [7] |
| Atg7-/- VSMCs | Mice | Atherosclerosis | Defective VSMC autophagy led to upregulation of MMP9, TGFB and CXCL12 | Promote postinjury neointima formation and diet-induced atherogenesis | [56] |
| Laminar flow | HUVEC | Atherosclerosis | Endothelial autophagy and SIRT1 expression induced contribute to the inhibition of Hippo/YAP signaling | Interrupte atherosclerotic plaque formation | [58] |
| Danqi pill | H9C2 cell | Heart failure | Via regulating the AMPK-TSC2-mTOR signaling pathway to restore autophagy | Improve cardiac function and protect against cardiomyocytes injury | [16] |
| Qi Dan Li Xin pill | Rat | Heart failure | Regulate mTOR/ p70S6k-mediated autophagy | Improve chronic heart failure | [55] |

Table4: Possible mechanisms of autophagy inducers in the treatment of cardiovascular diseases through up-regulation of autophagy. (Rheb: Ras homology enriched in brain, MiRNA-212/132: microRNA-212/132, DNase: Deoxyribonuclease, VSMCs: Vascular smooth muscle cells, ATG: Autophagy-related, MI: Myocardial infarction, mTORC1: mechanistic target of rapamycin complex 1, Foxo3: Forkhead box O3, DNAse: Deoxyribonuclease, VSMC: Vascular smooth muscle cell, TGFB: Transforming growth factor-beta, CXCL12: CXC chemokine ligand 12, SIRT1: Sirtuin-1, YAP: Yes-associated protein, mTOR: mechanistic target of rapamycin, TSC: Tuberous sclerosis, AMPK: AMP-activated protein kinase. )
