# Supplementary material for: Programmed Cell Death: Complex Regulatory Networks in Cardiovascular Disease
Source: Front Cell Dev Biol. 2021 Nov 26;9:794879. doi: 10.3389/fcell.2021.794879 (PMC8661013; doi:10.3389/fcell.2021.794879)
Supplement: Supplementary file 9 [file Table6.DOCX]

| Reagents | Materials | Diseases | Mechanisms | Effects | Reference |
| --- | --- | --- | --- | --- | --- |
| Long non-coding RNA Kcnq1ot1 | Mice | Diabetes cardiomyopathy | By Kcnq1ot1/miR-214-3p/caspase-1/TGF-β1/smads signal pathway | Improve cardiac function and fibrosis | [63] |
| Calpain silencing | Mice | I/R | Through the NLRP3/ASC/Caspase-1 axis | Alleviate I/R injury | [21] |
| MicroRNA-29a | Mice | I/R | By targeting SIRT1 and Suppressing Oxidative Stress and NLRP3-Mediated Pyroptosis Pathway | Alleviate I/R injury | [64] |
| Cytoprotective | Mice | I/R | Via mTORC1 inhibition | Activate protein C averts Nlrp3 inflammasome-induced I/R injury | [18] |
| Cathepsin B inhibition | Rat | MI | By inhibiting the NLRP3 pathway | Attenuate cardiac dysfunction and remodeling following MI | [65] |
| MicroRNA-9 | H9C2 cell | Diabetic cardiomyopathy | Inhibition of miR-9 upregulates ELAVL1 expression and activates caspase-1 | Attenuate hyperglycemia-induced ELAVL1 and inhibit cardiomyocyte pyroptosis to prevent cardiomyocyte cell loss in diabetics | [66] |
| MicroRNA-22 | Rat | Atherosclerosis | By targeting NLRP3 through suppression of the inflammasome signaling pathway in a rat model of coronary heart disease | Against endothelial cell injury | [67] |
| Melatonin | Mice | Atherosclerosis | Via regulation of long noncoding RNA MEG3/miR-223/NLRP3 axis | Prevent endothelial cell pyroptosis | [62] |
| MicroRNA-30c-5p | HAEC | Atherosclerosis | Through FOXO3 down-regulation in atherosclerosis. | Inhibit NLRP3 inflammasome-mediated endothelial cell pyroptosis | [68] |
| PEDF | Rat | Ischemic cardiomyocytes | Through PEDF receptor/phospholipase A2 | Inhibit the activation of NLRP3 inflammasome in hypoxia cardiomyocytes | [69] |
| VX-765 | Mice | Atherosclerosis | By modulating VSMCs pyroptosis | Attenuate atherosclerosis in ApoE deficient mice | [70] |

Table 6: Possible mechanisms of pyroptosis inducers regulating pyroptosis in the treatment of cardiovascular diseases. (PEDF: Pigment epithelium-derived factor, VX-765: caspase-1 inhibitor, MI: Myocardial infarction, I/R: Ischemia/reperfusion, TGF-β1: Transforming growth factor-β1, Nlrp3: NOD-like receptor 3, ASC: Apoptosis-associated speck-like protein containing a caspase recruitment domain, SIRT1: Sirtuin-1, mTORC1: mechanistic target of rapamycin complex 1, ELAVL1: ELAV like RNA binding protein 1, MEG3: Maternally expressed 3, VSMC: Vascular smooth muscle cell, Foxo3: Forkhead box O3, PEDF: Pigment-epithelium-derived factor, ApoE: Apolipoprotein E. )
