# Supplementary material for: Programmed Cell Death: Complex Regulatory Networks in Cardiovascular Disease
Source: Front Cell Dev Biol. 2021 Nov 26;9:794879. doi: 10.3389/fcell.2021.794879 (PMC8661013; doi:10.3389/fcell.2021.794879)
Supplement: Supplementary file 10 [file Table10.DOCX]

| Drug | Diseases | Trial  Phase | Sample  Size | Mechanisms | Effects | NCT | Reference |
| --- | --- | --- | --- | --- | --- | --- | --- |
| Berberine | Patients after PCI | Ⅱ | 100 | Through the AMPK/mTOR pathway | Reduce myocardial injury | CMU-201500605 | [24] |
| Simvastatin | Patients after extracorporeal circulation | Ⅱ | 130 | Reduce the expression of LC3-II/LC3-I and Beclin 1, and increase the expression of phosphorylation of AMPK | Reduce myocardial injury | ChiCTR-TRC-14005164 | [109] |

Table10: Clinical trials involving autophagy of the cardiovascular system. (PCI: percutaneous coronary intervention, AMPK/mTOR: AMP-activated protein kinase/mammalian target of rapamycin. )
